# Feasibility of Good Governance at Health Facilities: A Proposed Framework and its Application Using Empirical Insights From Kenya

**DOI:** 10.34172/ijhpm.2021.01

**Published:** 2021-01-23

**Authors:** Thidar Pyone, Tolib Mirzoev

**Affiliations:** ^1^Department of Global Public Health, Public Health England, London, United Kingdom.; ^2^Leeds Institute of Health Sciences, University of Leeds, United Kingdom.

**Keywords:** Framework, Health Systems Governance, Institutional Analysis Theory, Organisational Readiness, Development Studies, Kenya

## Abstract

**Background:** Governance is a social phenomenon which permeates throughout systemic, organisational and individual levels. Studies of health systems governance traditionally assessed performance of systems or organisations against principles of good governance. However, understanding key pre-conditions to embed good governance required for healthcare organisations is limited. We explore the feasibility of embedding good governance at healthcare facilities in Kenya.

**Methods:** Our conceptualisation of organisational readiness for embedding good governance stems from a theory of institutional analysis and frameworks for understanding organisational readiness for change. Four inter-related constructs underpin to embed good governance: (*i*) individual motivations, determined by (*ii*) mechanisms for encouraging adherence to good governance through (*iii*) organisation’s institutional arrangements, all within (*iv*) a wider context. We propose a framework, validated through qualitative methods and collected through 39 semi-structured interviews with healthcare providers, county and national-level policy-makers in Kenya. Data was analysed using framework approach, guided by the four constructs of the theoretical framework. We explored each construct in relation to three key principles of good governance: accountability, participation and transparency of information.

**Results:** Embedding good governance in healthcare organisations in Kenya is influenced by political and socio-cultural contexts. Individual motivations were a critical element of self-enforcement to embed principles of good governance by healthcare providers within their facilities. Healthcare providers possess strong moral incentives to self-enforce accountability to local populations, but their participation in decision-making was limited. Health facilities lacked effective mechanisms for enforcing good governance such as combating corruption, which led to a proliferation of informal institutional arrangements.

**Conclusion:** Organisational readiness for good governance is context-specific so future work should recognise different interpretations of acceptable degrees of transparency, accountability and participation. While good governance involves collective social action, organisational readiness relies on individual choices and decisions within the context of organisational rules and cultural and historical environments.

## Background

**Key Messages**

** Implications for policy makers**

- Good governance is a key component of well-functioning national health systems. Yet, it remains an abstract concept, and there are no frameworks for organisational readiness to embed principles of good governance, perhaps reflecting the complexity of the abstract concept of governance. Our empirical results from Kenya suggest that decision-makers need to particularly consider individual motivations of healthcare providers to embed principles of good governance within their facilities.
- Appropriate institutional arrangements, including enforcement of formal and informal rules alongside their effects on individual motivations, are critical for embedding principles of good governance within healthcare organisations.
- A useful framework is proposed for understanding and assessing organisational readiness for embedding principles of good governance. The proposed framework can be applied to national health programmes, different health areas and specific healthcare facilities.

** Implications for the public**

Good governance is a key component of a well-functioning health system, which require (1) motivations from individuals within organisations; (2) favourable organisational arrangements; (3) mechanisms enforcing those arrangements; within (4) a wider health system, political, socio-economic context.

Good governance, a phenomenon which involves a balance between technical and social dimensions and permeates throughout systemic, organisational and individual levels, is a key component of effective national health systems.^1,2^ Good governance is defined as “*developing and setting effective rules in the institutional arenas for policies, programmes and activities relevant to fulfil public health functions to achieve the objectives of the health sector*.”^3^ The World Health Organization (WHO) defined governance alongside leadership as “*ensuring strategic policy frameworks exist and are combined with effective oversight, coalition building, attention to system design and accountability*.”^4^

 Governance is a key determinant of overall health systems performance.^5^ The importance of good governance is also reflected in the Sustainable Development Goals as Goal 16, comprising the rule of law, accountability, participation and transparency. More recently, the global “Health Systems Governance Collaborative” was officially launched in December 2016 to capitalise approaches to health systems governance through collective efforts (https://hsgovcollab.org/).

 Despite its recognised importance, research in health systems governance is often neglected, and many perceive governance as a rather abstract concept.^6^ Consequently, research in governance does not always result in an actionable agenda to inform and support actions by health policy-makers and practitioners.^7^ Multiple theoretical frameworks and subsequent empirical assessments of health systems governance are becoming increasingly available,^8^ and locus largely on assessing the degree of good governance within systems or institutions. However, little attention is paid to understanding key pre-conditions for embedding principles of good governance within healthcare organisations.

 In this paper, we attempt to bridge this knowledge gap by advancing the understanding of the organisational readiness for embedding principles of good governance. We draw upon insights from two theoretical disciplines: development studies and institutional economics^9^ and utilise understanding of organisational readiness for change in areas of knowledge translation, action research or acute stroke.^10-13^ To illustrate its practical applicability, the proposed framework is validated in the assessment of readiness for good governance of maternal and newborn healthcare facilities in Kenya.

 This paper aims to answer the following research question: what are the key pre-conditions for organisational readiness for good governance within healthcare organisations? Our intention is not to add to the plethora of frameworks on health systems governance, but to inform theoretical debate and future empirical work to embed good governance within healthcare organisations.

 The paper is structured as follows. After description of the methods, we summarise the underlying conceptualisations of governance to introduce and explain our theoretical framework. The framework is used to explore organisational readiness for good governance in a case study from Kenya. Finally, we reflect on the possible implications for future scholarship on this topic.

## Methods

 The proposed framework stems from the systematic literature review of governance^8^ and empirical assessments of implications of free maternity services on health systems governance in Kenya,^14^ both summarised in Table 1. It drew on the institutional analysis theory and organisational readiness,^10-13^ which are set out later in explaining our framework.

**Table 1:** Steps in Developing Framework for Organisational Readiness for Good Governance

**Steps**	**Methods Used**	**Inputs Into the Framework**
1. Systematic literature review	Mapping of existing frameworks to assess health systems governance Identification of theory and framework for health systems governance assessment in Kenya	Conceptual and theoretical inputs to understanding health systems governance
2. Health systems governance assessment in Kenya	Semi-structured interviews with key informants in Kenya Analysing data on free maternity services policy using the framework identified in Step 1	Empirical assessment of health systems governance within Kenyan context
3. Adapt and test the revised framework	Adapting the framework used in Step 2 through incorporating concepts of organisational readiness Re-analyse Kenyan data to test the framework	Conceptual and theoretical inputs Framework design and content

 During Step 1, a systematic review was conducted, with methodology and results available elsewhere (see Pyone et al^8^). It reviewed theoretical underpinnings of health systems governance and analysed frameworks for assessing governance. From a total of 16 frameworks, two utilised the institutional analysis theory, which is of particular relevance to this paper. The review also did not reveal any models that explain organisational readiness for good governance within healthcare organisations. In Step 2, an empirical assessment of health systems governance was conducted in Kenya, based on qualitative data from semi-structured interviews with purposively-selected healthcare workers, county health officials, and health policy-makers. Results of general governance assessments in relation to free maternity services policy are available elsewhere (see Pyone et al^14^).

 The Step 3 is the main focus of this paper. The proposed framework for organisational readiness for good governance was informed by our understanding of good governance from previous papers. The systematic review in Step 1 highlighted gaps in knowledge on organisational readiness for good governance. Therefore, we conducted a scoping review on organisational readiness for change. We searched for published literature in English, using keywords such as “organisation/organisation,” “organisation*/organisation* change,” “organisation*/organisation* readiness” in PubMed, Google and Google Scholar. Papers that discuss specifically organisational readiness or its related concepts within the health and healthcare domain were included. Reference search of relevant, included papers was also conducted. This was followed by a series of discussions amongst the authors which involved initial reflections on the conceptual understanding of organisational readiness in the reviewed literature, followed by thematic analysis and then subsequent categorisation of emerging constructs into a coherent framework introduced in the Results.

 For thematic analysis, qualitative data from semi-structured interviews conducted in Kenya were then re-analysed using the new lenses on organisational readiness for good governance, to test the applicability and validity of the proposed framework. No further data collection was conducted, because the previously collected dataset during Step 2 was deemed rich enough to yield sufficient insight into the organisational readiness for embedding good governance within Kenyan healthcare facilities. Our focus on Kenya was due to an on-going decentralization, which positioned this study to provide timely lessons for policy and practice. Maternal and newborn health facilities were chosen because maternal and newborn health is one of the key priorities in Kenya; and health workers at operational level could provide useful justifications to illustrate the study. Table 2 describes key characteristics of participants. All interviews lasted approximately 30-90 minutes, were conducted in English at participants’ workplaces following obtaining of informed consent. Interviews were guided by the topic guide structured around accountability, participation, transparency of information and were audio-recorded for analysis. All data was analysed using Framework Approach,^15^ guided by the four constructs of our theoretical framework.

**Table 2:** Characteristics of Respondents Who Participated in the Study

**Health System Level**	**Institution**	**Participant**	**Participated (n = 39)**	**Invited (n = 43)**
National (policy)	Ministry of Health; multilateral and bilateral organisations	Senior directors and advisors	10	11
Country (policy)	County Health Department	Chief Officer of Health	5	6
		County Director of Health	5	6
Facility (implementation)	Government health facilities offering comprehensive maternity care	Doctor in-charge	10	10
		Nurse in-charge of maternity	9	10

Source: Pyone et al.^14^

## Results

###  Organisational Readiness for Good Governance Framework

 From the systematic review in Step 1, two frameworks for understanding governance are of relevance to this paper. They are Baez-Camargo and Jacobs^16^ and Siddiqi et al.^17^ The ‘inputs-processes-outputs’ framework by Baez-Camargo and Jacobs takes an exogenous view of institutions by asserting the causal links between the institutional arrangements (governance inputs and processes) and the governance-related system outcomes.^18^ The framework by Siddiqi et al distinguishes ten principles of good governance, structured under three domains: context, processes and outcomes and disaggregated into 63 questions. Siddiqi et al argue that their qualitative framework can be used to compare governance functions across different contexts and to assess governance at both national and sub-national levels.^17^

 Both of these frameworks stem from the Douglas North’s theory of institutional analysis in which institutions are understood “*as humanly devised constraints that shape human interactions*.”^19^ This definition comprehensively covers both macro (contextual) and micro (individual) levels, allowing for both formal processes and informal customs.^18^ Such an approach to understanding governance is increasingly considered to be most effective as this enriches the understanding of practical challenges encountered to embed governance.^7,9^ Both frameworks have been used in assessments of health systems governance at policy formulation and implementation levels. These frameworks embed the selected principles of governance within formal and informal constraints that shape an organisation’s functions. The most commonly referred to principle appears to be accountability, followed by control of corruption, transparency, participation and inclusiveness.^8,20,21^

 Organisational readiness is a multi-level and multi-faceted construct.^12,22^ Adopting the concept of organisational readiness ensures basic elements to assess readiness on the change process, content, context and the reactions.^22^ Weiner defined organisational readiness as “*organisational members’ change commitment and change efficacy to implement organisational change*.”^23^ In this paper, we define organisational readiness as a commitment from health facility staff to embed principles of good health systems governance through becoming more accountable, transparent and inclusive in their decision-making.

 Two theoretical considerations inform the understanding of organisational readiness: psychological aspect (attitudes, beliefs and intentions of members of an organisation) and structural aspect (organisational resources and organisational capacities).^24^ In other words, the notion of organisational readiness comprises both organisational and individual levels.^24^ These considerations guide the application of the concept of organisational readiness for change in areas such as knowledge uptake and translation, conducting action research or implementing acute stroke care.^10-13^ These two broad considerations inform five detailed constructs which are evident in most frameworks of organisational readiness for change: motivation, resources, organisational climate, staff attributes and change processes.^23-26^

 While the institutional analysis theory highlights the micro (individual) and macro (systems) levels of governance, the literature on organisational readiness helps to usefully unpack the meso (organisational) level which is the focus of the proposed framework.

 Our theoretisation, shown in Figure, embeds conventional principles of good governance (from development studies) and understanding of governance as a collective action (from the institutional analysis theory), within five key constructs from the organisational readiness literature.

**Figure:**
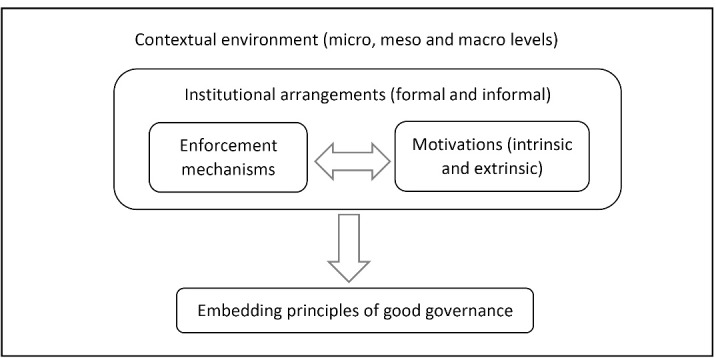
Framework for Organisational Readiness for Good Governance.

 The core of our framework is a set of pre-conditions which shape an organisation’s readiness for embedding principles of good governance. Our argument is that embedding key principles of good governance within healthcare organisations are subject to appropriate institutional arrangements which enable enforcement of new practices and motivate individuals to adhere to these changes. A key concept is that individuals within the organisations interact to achieve a collective effort reflected in the adoption of good governance. Any individual choices and decisions are made within the context of organisational rules and within cultural and historical contexts of their environments.

 Embedding principles of good governance is shaped by four inter-related constructs (Table 3): (*i*) individual motivation, which is determined by; (*ii*) mechanisms for enforcement of and adherence to principles of good governance, through; (*iii*) the organisation’s institutional arrangements and within; (*iv*) a wider contextual environment. The individual’s motivation is a crucial determinant of healthcare providers’ and policy-makers’ willingness to subscribe to any new initiative to embed good governance. Organisational enforcement mechanisms affect individual motivations through a combination of incentives and sanctions. These mechanisms are shaped by formal and informal rules, which constitute institutional arrangements and shape the collective efforts of staff within organisations. The contextual environment is intentionally shown as an overarching construct which influences all other components of the framework.

**Table 3:** Key Constructs of the Framework

**Construct **	**Description**
1. Individual motivation	Determined by different incentives which (de)motivate individuals to adhere to principles of good governance Intrinsic motivation is when an individual values the change Extrinsic motivation is when an individual expects rewards from an organisation for adhering to good governance
2. Enforcement mechanisms	Organisational processes and mechanisms designed to ensure that individuals within the organisation adhere to good governance Self-enforcement, following intrinsic motivation Third-party enforcement, through organisational rules
3. Institutional arrangements	Rules of the organisations (for each principle of governance - accountability, participation, transparency of information) Formal rules (eg, constitutions, contracts, property rights) Informal rules (behavioural norms, code of conduct)
4.Context	Socio-economic, political or resource environment which influences behaviour of individuals within an organisation, comprising: Micro (individual) level Meso (organisational) level Macro (systems) level

 Motivation can be intrinsic, ie, when a healthcare provider adheres to the facility arrangements as s/he believes in and values it, and extrinsic, ie, when a health provider expects to benefit something by adhering to principles of good governance.^27^ Examples of intrinsic motivators include belief in equity and social justice, whereas an example of extrinsic motivator can be the expectation of bonus payments for good performance or administrative sanctions for non-adherence.^1^ These two types of motivations are closely intertwined and can be difficult to fully differentiate.^14,27^ Consequently, combinations of intrinsic and extrinsic motivators will be required to catalyse decisions on whether or not to embed new practices (such as principles of good governance) across the different individuals. The degree of individual motivation is shaped by different incentives available to healthcare staff to embed good governance and consequently “*…ensure responsive, effective and efficient services*.”^28^ These incentives include monetary and non-monetary benefits accrued through institutional arrangements.^27^

 Enforcement mechanisms comprise organisational processes and mechanisms which can ensure that individuals and teams adhere to good governance.^27,29^ Enforcement can occur via self-enforcement by the individuals and small groups based on common beliefs, taking into account reputation and kinship; and third-party enforcement through legal sanctions, contracts, rules, laws, or policing.^19^ Both types are complementary, and each will be required to embed organisational change. The optimal balance between ‘the carrot and the stick’ in relation to enforcement mechanisms will be specific to each organisation’s culture and context and to the individual health service providers.^30^ Ultimately, a combination of self- and third-party enforcement is likely to trigger intrinsic and extrinsic motivations and catalyse the individual’s decision whether to subscribe to the principles of good governance.

 North defined institutions as “*the rules of the game*” and organisations as “*the players of the game*.”^19^ All organisations comprise formal rules (institutional arrangements) such as political or legal constitutions and contracts; and informal rules such as codes of conduct, behavioural expectations and conventions.^19,29^ Any actions and decisions by, and relations between, the individuals within an organisation are enabled or constrained by the inter-related formal and informal rules. A combination of formal and informal rules is likely to influence to embed good governance within healthcare organisations.

 The context is understood as comprising a variety of influences including socio-economic, political and resource environments (resource availability or flexibility in use of resources including physical and human resources).^31^ It is an important determinant of health systems governance as it affects decision-making through a multitude of influences permeating through interrelated individual (micro), organisational (meso) and systems (macro) levels.^32^ An in-depth understanding of contextual influences across the micro-meso-macro levels helps to better recognise the complexity of multiple triggers of specific mechanisms leading to the desired or undesired outcomes.^33^ A combination of factors at all levels is required to catalyse the intrinsic and extrinsic motivations of individuals to subscribe to principles of good governance.

 The four constructs are inter-related, and altogether they determine the organisation’s ability to introduce principles of good governance. For example, a combination of monetary and non-monetary incentives, supported by adequate enforcement mechanisms within a favourable facility and systems environments comprising participatory, accountable, transparent and informed decision-making, can motivate staff to embed principles of good governance in their organisations.

###  Framework Application on a Case Study From Kenya 

 In this section, we explore the utility of the proposed framework through applying it to the context of maternal and newborn healthcare facilities in Kenya. We explore each construct in relation to three key principles of good governance: accountability, participation and transparency of information. Accountability is defined, in the traditional sense, as the obligation to accept responsibility for one’s actions. Initially, it was a synonym for “citizen’s voice,” though more recently accountability is related to a relationship between people, policy-makers and providers.^7,8,34^ The participation refers to actors’ engagements in decision-making, and by transparency of information we mean the degree to which information is openly shared for informing decisions.

 To put this research in context, the data for this study was collected in 2015, two years after devolution. Simultaneously with devolution, in June 2013, the government introduced two prominent health financing initiatives, the abolishment of user fees policy and the adoption of the free maternity services policy.

####  (i) Individual Motivation

 Healthcare workers shared a view that it is their moral mission and ethical obligation to provide appropriate treatment to the people they serve. They highly valued the humanitarian aspect of their work, particularly their role in saving lives. This passion was one of their key intrinsic motivators, and despite limited resources, they felt satisfied that their services still benefit women and children. Such intrinsic motivators made them feel accountable for their work. Despite the lack of resources to perform emergency obstetric care (the equipment and trained health staff to perform signal functions), healthcare providers continued to provide services. However, some healthcare providers were reportedly demotivated due to lack of opportunities to participate in policy-making. There was a perception that they were left out of, and therefore lacked voice in the policy development process:

 “*The government pegged 5000sh for delivery, and they did not know that there is a difference between normal delivery and a CS … They should have said if you deliver normal, we give you 5000sh. If you deliver CS, we give you 20 000sh because the two are different, the cost of anaesthesia is different from normal delivery … We were not called to craft the payment structure; we were not consulted, it was just pushed down our throat that you will get 5000 and you cannot say no because it is the government, so you just work with what you have*” (KI-16, Health worker).

 A key prerequisite for the motivation of healthcare providers was a fulfilment of their basic needs, which has not always been the case. For example, they did not receive their salaries on time sometimes “for months” with no information, and staff in some facilities felt insecure in their jobs as there were rumours regarding laying off health staff.

 “*There has been a lot of issues concerning salaries, and some county says they want to lay off some people. Some people salaries have not been paid for months, some people have not been promoted, and you see there is a lot of hardship among the people who provide care in the hospital*” (KI-38, Health worker).

 Supportive working environment, presence of a facility-driven staff supports system and collaboration with colleagues were also particularly emphasised as important extrinsic motivators for embedding principles of good governance.

####  (ii) Enforcement Mechanisms

 Facilities set up organisational accountability processes after agreement with the county department of health to control embezzlement. For instance, some facilities set up facility-driven disciplinary actions to control corruption. Healthcare providers demonstrated self-initiative in performing their duties as they improvised with the available resources to provide health services even though the equipment and supplies required to perform certain procedures were insufficient.

 When individuals did not have means and motivation to self-enforce organisational rules, third party enforcement became essential as it can enable making things happen. In the following example, a health facility has suggestion boxes to improve services provided as a means of accountability. However, without proper enforcement (both self-enforcement and third-party enforcement), having suggestion boxes are not useful.

*“We have two or three suggestion boxes in the hospital that don’t even function…The boxes are just installed somewhere. But checking whether there is anything inside or who is responsible for these, we don’t know. They are just there”* (KI-37, Health worker).

 Participants from health facility and national levels highlighted the importance of third-party enforcements to control corruption as a means to promote accountability. All shared a view that accountability across all sectors, including health, require improvement. Most participants shared a view that control measures for corruption were not enforced within the health system. The existing regulatory system was reportedly weak as “*a lot of people tend to think healthcare as a business*.” Readiness to fight corruption among different organisations varies as cross-sectoral actions are required, and a need to go beyond the health sector as corruption can be systemic:

 “*I believe corruption or money can take anybody anywhere. Some of these things I am sure they are bought. They are bought as when you want a permit to run maternity; if it has a requirement that you should have qualifications for you to run maternity, then what if you give them money, these people may protect you*” (KI-9, Health worker).

 County officials noted staff supervision as the most common form of third-party enforcement. Healthcare providers felt supported during those supervisory visits, which allowed them to interact with their supervisors and learn from those interactions. Interview participants also felt that supervision also contributed to healthcare providers’ willingness and readiness to sustain the transparent dialogue on their performance and become more accountable for their clinical duties.

####  (ii) Institutional Arrangements

 Formal institutional arrangements to improve organisational accountability, such as staff performance appraisal system did not exist. Interview participants from different health system levels commented about the lack of an objective system of appraisal to improve the performance of public sector staff. In the recently-devolved health system, staff promotions were managed at the county level and healthcare providers that “*they [the county] didn’t know who was doing what at the facility, so it was just the system that after every three years you get promoted*.” Hence, healthcare providers felt demotivated due to ‘stagnation’ and the lack of opportunity to progress in the role. They also felt that authorities who were new to their positions did not understand the health system:

*“The reward system is rather demotivating. Because you find someone has worked for a long time, somebody works very well, and you find she has stagnated all years, she has stagnated in one job group … So, these are things which need to be streamlined because now if you are to wait for the public service commission to create positions, how would the public commission know that people are stagnating unless they come to the ground*” (KI-19, Health worker).

 Informal institutional arrangements emerged when there were gaps in the formal arrangements for rewards or performance appraisal for accountability. Facilities were ready to set up their own form of informal appraisal mostly at their own discretion such as organising annual staff parties, sending staff to a conference or workshop, awarding certificates or trophies, or words of appreciation from the managers. This also contributed to intrinsic and extrinsic motivations and served as intra-facility enforcement mechanisms of good performance.

 Despite the presence of formal rules and procedures to discipline public health facility staff, there was a reluctance to do so when the organisations were not ready to introduce greater accountability and transparency. It was noted that some healthcare providers were exempt from “*punishment*” (which should have been provided) which further “*discouraged from disciplining another person*” in the future.

 “*I have never seen anybody going home because of a problem. Instead, they are transferred to another place. So, you transfer the problem to another place. Because there was another relationship with the accused, whether you are relative, or you are relative to their husband or something like that. You never know you can be compromised. Also, we are human beings. So, you have done something like an incentive; action is not taken by your senior. As a result, you get discouraged from disciplining another person next time*” (KI-9, Health worker).

 In such organisational culture, people took advantage of the situation where the procedure to dismiss them was long and complicated and no action could be taken against them even if an individual staff committed some form of embezzlement. One healthcare provider recounted how the hospital management weakened the proposal of disciplinary action due to the fear of potential consequences or backlash:

 “*We may recommend at the lower level, but at the higher level, it may be disapproved … We kept quiet… The backlash will be there. That is called impunity, will now start doing things with impunity that can happen. Well, I may mention only one … a certain doctor has failed to come for duty, and in the event, a patient has died because there was no doctor to attend. Then, we do a disciplinary committee and recommend that doctor’s license be suspended, and the recommendation goes higher, and they refuse to suspend his license. It worked negatively for the hospital*” (KI-16, Health worker).

####  (iv) Contextual Environment

 Participants from national and county levels felt that devolution provided opportunities for improving participatory local decision-making as it created opportunities to identify local needs and facilitate policy discussions among local government and populations, ultimately contributing to improve accountability of health facilities to the local populations. Devolution reduced the previously lengthy decision-making processes and provided opportunities for clear feedback mechanisms at lower (local) levels. Devolution increased opportunities for more efficient local decision=making, by “*bringing the person closer to the decision-making, closer to the action*.” In doing so, county-level participants believed this had increased responsiveness to local needs, which would otherwise be difficult to identify from the national government level. One county official reflected that:

 “*Since devolution, we were able to implement that very effectively. We are making our decision at this lower level, so it is not very hard for us. Because we get our own funds at the county level, we can do our own supervision in our own time frame*” (KI-35, a county official).

 Frequent staff rotations and transfers are still health system “bottlenecks” which can significantly undermine healthcare providers’ accountability and productivity.

 “*Emergency Obstetric Care, as far as the training is concerned, there are still many people who have not been trained; there are about less than 50% who have been trained. But you see we are having continuous transfers; so you train somebody who is transferred and takes that knowledge to somewhere else. So not many people have been trained, and also, people go for the training, and you are not in the department where you can practice, then anything that is not used is wasted, isn’t it*?” (KI-9, Health worker).

 Many participants were keen to emphasise that inadequate health financing had been a critical stumbling block to health system progress and caused the health system to become vulnerable to embezzlement. Healthcare providers highlighted that despite the free maternity services policy in place, user fees still exist, stressing that “*patients have to pay*” and “*there is no free medication*.” When health facilities had challenges in adhering to the free maternity services policy due to the complicated nature of the refund mechanism, resulting in delayed disbursements from national to county level. This forced public health facilities to charge maternity services which were supposed to be free. It highlights an easy opportunity for corruption within ineffective institutional arrangements which counter-balanced the motivations and self-enforcement of accountability. Table 4 summarises key findings in relation to each construct.

**Table 4:** Key Findings of the Framework in a Case Study From Kenya

**Constructs of the framework**	**Key Findings**
**i. Individual Motivation**	
Intrinsic motivation	- Healthcare providers demonstrate a moral mission to adhere to good governance such as being accountable for their jobs and feeling satisfied for helping to save lives. - Healthcare providers were demotivated due to lack of opportunities to participate in the policy-making process.
Extrinsic motivation	- The need to meet basic needs such as receiving regular salaries and a supportive working environment, the presence of a facility-driven staff support system and collaboration/coordination among colleagues to adhere to principles of good governance. - Availability of competent staff to effectively manage and govern.
**ii. Enforcement Mechanisms**	
Self-enforcement	- Improvisation using available resources because healthcare providers felt accountable. - Healthcare facilities set up their own form of enforcement with the county department to control embezzlement. - Self-enforcement was noted to be important in remote facilities where third-party supervision is a challenge.
Third party enforcement	- Supervision and oversight mechanisms by county or state officials. -A lack of enforcement of the regulatory system was documented. - Corruption was reported as being systemic, and hence, the cross-sectoral action is essential with enforcement from an independent third-party.
**iii. Institutional Arrangements**	
Formal	- There is a lack of formal mechanisms to improve accountability of public health staff, lack of clear lines of accountability, standard operating procedures and protocols. - According to the new Constitution, it is a legal requirement that citizens must be consulted before a policy can be enacted into law.
Informal	- County governments and healthcare facility in-charges set up their own forms of (informal) appraisal to improve staff accountability and at their own discretion. - It was noted that some healthcare providers were exempt from “*punishment*” (which should have been provided) which further “*discouraged from disciplining another person*” in future. - Healthcare facilities are subject to “top-down” or “county-centric” policy decisions which constrain local participation in decision-making. - A general lack of transparency and absence of structured information dissemination was reported.
**iv. Contextual Environment**	
Devolution Political, socio-cultural context Health financing	- A shift from a centralised to a decentralised health system, through the transfer of responsibilities to county governments and the way healthcare facilities are governed, was implemented. - Inadequate health financing had been a critical stumbling block to health system progress and caused the health system to become vulnerable to embezzlement. - Frequent staff rotation and staff transfer are still health system “bottlenecks” and also undermine healthcare providers’ accountability and productivity. - Human resources for health. - Medicines, supplies and equipment. - Lack of medicines and supplies compromised healthcare providers’ accountability under the policy of free maternity services policy as they had to write prescriptions.

## Discussion

 In this paper, we have examined organisational readiness of healthcare facilities in Kenya for embedding three key principles of good governance.

 Regarding the accountability principle, individual healthcare providers are motivated, though somewhat hindered by devolution challenges and not receiving regular salaries. Nevertheless, stakeholders looked for ways to enforce accountability arrangements. The framework spotlights gaps in formal institutional arrangements to improve performance accountability, such as performance appraisal for health staff. Alande presented a similar finding in Mombasa district that human resource development was a neglected area, as there was no system for developing county officers to further the agenda for devolution.^35^ Therefore, the framework highlights how organisational arrangements and wider socio-political context complement individual motivations. Similarly, Booth and Cammack underscored that social accountability can be effective together with other complementary mechanisms in place, such as top-down pressure, opportunities to integrate social movements into political parties, and interest from professional organisations.^36^

 In regards to inclusive participation, the framework highlights a discrepancy between the actual implementation of the formal institutional arrangements for participation as healthcare providers complaint of their missed opportunities to participate in policy-making processes. Other studies in Kenya observed similar findings.^37,38^ In the study of Lipsky et al, the authors did not observe any involvement by citizens or civil society organisations in county policy and programme selection processes.^37^ Similarly, Barasa et al and Molyneux et al did not observe participation and transparency in budgeting and priority settings in their case study hospitals.^38^ Indeed, although county participants mentioned that they had public hearings or barazas where the public could provide suggestions to the country government on a proposed policy, actual public opportunities to provide such recommendations were varied. Among three counties studied by Lipsky et al, only one county held such an event to receive feedback from citizens, while in another county, a single official developed the health sector budget without additional input.^37^

 In relation to transparency of information, individual healthcare providers were demotivated as there was a perception that staff promotions were handled without transparency and without a policy. Devolution was reported as an opportunity because county governments could release information tailored to their local needs. This could be enforced by regular and timely notification to healthcare providers to keep them engaged in policy-making processes. Empirical results from Kenya underscore important lessons on organisational readiness for good governance. The results highlight that while organisational arrangements and enforcement mechanisisms are important to embed good governance, it is essential to recognise intrinsic and extrinsic motivators of individuals from the organisations. All these interplayed in a wider context, including health system, political and socio-economic.

 Current frameworks for understanding organisational readiness for change typically comprise five key constructs (motivation, resources, organisational climate, staff attributes and change process) explored from two aspects (psychological and structural).^23-26^ While three of the four constructs within our framework reflect these constructs, the construct of “institutional arrangements” represents a combination of change processes within the organisational climate to encourage adherence to the principles of good governance. The inclusion of institutional arrangements reflects the North’s institutional analysis approach used in our framework, and also makes our framework more governance-focused by emphasising readiness for embedding principles of good governance as compared with more general change.

 Each of the four constructs of the proposed framework (individual motivations, enforcement mechanism, institutional arrangements and context) are important in their own right and allow for assessing readiness for good governance at healthcare facilities. They are clearly inter-related and should be taken together as a framework, for example absence of punishment being a motivator for embezzlement, shaping the proliferation of informal institutional arrangements and reflecting the wider contextual environment.

 The proposed framework is not specifically related to any specific principles of governance, although, in our case study, we assessed accountability, inclusive participation and transparency of information. As Bigdeli et al highlighted in their revised health systems governance framework, governance arrangements exist both between and within three stakeholder groups (people, healthcare providers and policy-makers).^7^ Our framework cuts across all levels and hence, generic and not limited to any principles. Therefore, this framework can be used to any principles of governance after being adapted to relevant organisational arrangements. A caveat is appropriate here that even though we have prioritised three key principles of good governance, the choice and application of principles of good governance should be cognisant of specific micro, meso, and macro contexts.^17,39^

 Consequently, recent literature on health systems governance refers less to a fixed set of principles of good governance that are required, and more to governance as a process of collective action through continuous interactions between different health systems actors.^7,1,8^ This can be explained by two reasons. First, different governance arrangements can have different degrees of effectiveness on embedding good governance – for example, appropriate enforcement mechanisms can facilitate motivation, but inappropriate mechanisms may lead to staff resentment.^40^ Second, care should be taken in gradually embedding good goverance in order not to hinder performance of existing processes – for example losses in efficiency can be due to successive and iterative consultations to ensure actors’ participation.^40^

 The fundamental conceptualisation of governance in new institutional economics defines it as a “collective action.” Likewise, organisational readiness is a “*shared property*” or “*shared psychological state in which team members are committed to organisational readiness for change and confident in their collective abilities to do so*.”^23^ This notion of collective action and shared value provided a common theoretical platform for synthesising the two bodies of literature in our conceptualisation of organisational readiness for good governance. Structural constructs such as enforcement mechanisms and institutional arrangements constitute key attributes of organisational readiness to introduce good governance, building on and extending the understanding of structural factors as a capacity to implement change in the literature on organisational readiness.^23,41,42^ However, our framework focuses specifically on prerequisites for embedding good governance. Such an approach provides a conceptually different angle to that of literature on organisational readiness which focuses primarily on the “outcomes” of the organisational change as a least studied area.^23^ The organisational readiness frameworks have set outcomes or targets that they want to achieve as a result of the organisational change. Most of them are in the form of some tangible results such as improvement in the quality of care, safety or efficiency.^23^ Though an equivalent set of outcomes in our framework are principles of good governance, our primary focus remains on key pre-conditions for embedding good governance in healthcare organisations.

 We illustrated the application of this framework on three key governance principles. The framework focuses directly on the governance being a social construct/phenomenon and permeating through different levels. In this paper, we have shown how a theoretical framework can be applied to assess organisational readiness for good governance of maternal and newborn healthcare facilities in Kenya in the context of devolution to identify potential supportive or inhibitory elements, which could assist to improve governance. We have intentionally applied our framework to local-level facilities in Kenya, to show that governance is not just a macro-level concept but can be a tangible phenomenon at a grassroots level. However, the proposed framework can also be used to assess organisational readiness within other health areas and different types of healthcare organisations, including national health programmes. For example, we envisage its applicability at both the organisational and system levels, ie, comprising multiple organisations.

###  Study Limitations

 We acknowledge a number of potential limitations of our framework. First, the North’s theory which underpins our framework is inherently vague in its nature.^18^ While we describe the four constructs of our proposed framework, they require adaptation to specific country contexts and/or health facility settings. Arguably, the need for further adaptation provides flexibility to inform a wider applicability of our framework. Second, we did not engage in the detailed debate as to whether organisational readiness varies in relation to embed individual principles of good governance. This was outside the scope of this paper, and further applications of the proposed framework can usefully explore this angle. Third, in our framework, we relied extensively on the qualitative assessment of organisational readiness for good governance. Qualitative methods do not always allow for the findings to be easily generalised across different settings, although their strengths include potential deeper explorations within their own contexts. Fourth, interviews were embedded in the validation process of the framework as we re-analysed the Kenyan data using the newly-developed framework instead of collecting new empirical data. We felt the existing dataset allowed us to explore the utility of the framework, which was its key purpose. Further empirical research of organisational readiness for good governance, including community participants, is needed, which can also usefully explore further adaptations of the proposed framework.

## Conclusion

 We gained deeper insights into the role of organisational arrangements to embed principles of good governance, drawing on different theories and academic disciplines. The proposed framework should inform structured analyses of a governance concept that is often seen as being abstract and diffuse.

 Macro-level devolution in Kenya has effected the meso-level organisational arrangements of healthcare facilities, influencing the way that individual healthcare workers responded in their daily lives. These enforce or inhibit the principles of good governance. It is imperative that policy-makers to consider institutional arrangements that enforce them to embed principles of good governance within health facilities.

 Understanding organisational conditions to embed principles of good governance should provide better understanding of how health systems function at the operational level and further theoretical debate and empirical assessments of organisational readiness for good governance is required.

## Ethical issues

 Ethical approvals for the whole study were obtained from the Institutional Ethics Review Committee at the Liverpool School of Tropical Medicine (reference 14.052) and the Kenyatta National Hospital-University of Nairobi Ethics Research Committee (reference KNH-ERC/A/98). Key ethical issues considered during the data collection and analysis included protecting respondents’ anonymity, data confidentiality, and ensuring that informed consent was obtained prior to every interview.

## Competing interests

 Authors declare that they have no competing interests.

## Authors’ contributions

 TP and TM conceived the idea for the paper. TP developed the tools, collected, analysed and interpreted the data. TP wrote the first draft of the manuscript. TP and TM critiqued all versions of the manuscript. Both authors read and approved the final version of the manuscript.

## Funding

 The Department of International Development UK provided funding for this research under the Making it Happen project (202945-101) between 2015-2017.

## References

[1] Gilson L, Lehmann U, Schneider H (2017). Practicing governance towards equity in health systems: LMIC perspectives and experience. Int J Equity Health.

[2] Gilson L, Doherty J, Loewenson R, Francis V. Challenging Inequity Through Health Systems. Final Report of the Knowledge Network on Health Systems. http://www.who.int/social_determinants/resources/csdh_media/hskn_final_2007_en.pdf. Published 2007.

[3] Brinkerhoff DW, Bossert TJ (2014). Health governance: principal-agent linkages and health system strengthening. Health Policy Plan.

[4] World Health Organization (WHO). Everybody’s Business--Strengthening Health Systems to Improve Health Outcomes: WHO’s Framework for Action. Geneva: WHO; 2007.

[5] Fryatt R, Bennett S, Soucat A (2017). Health sector governance: should we be investing more?. BMJ Glob Health.

[6] Loewenson R. Neglected Health Systems Research: Governance and Accountability [Technical Brief]. Geneva: Alliance for Health Policy and Systems Research, WHO; 2008. http://www.who.int/alliance-hpsr/resources/AllianceHPSR_ResearchIssue_Governance.pdf.

[7] Bigdeli M, Rouffy B, Lane BD, Schmets G, Soucat A. Health systems governance: the missing links. BMJ Glob Health 2020;5(8). 10.1136/bmjgh-2020-002533. PMC7422628 32784214

[8] Pyone T, Smith H, van den Broek N (2017). Frameworks to assess health systems governance: a systematic review. Health Policy Plan.

[9] Chhotray V, Stoker G. Governance Theory and Practice: A Cross-Disciplinary Approach. London: Palgrave Macmillan; 2008.

[10] Helfrich CD, Li YF, Sharp ND, Sales AE (2009). Organizational readiness to change assessment (ORCA): development of an instrument based on the Promoting Action on Research in Health Services (PARIHS) framework. Implement Sci.

[11] Gagnon MP, Attieh R, Dunn S (2018). Development and content validation of a transcultural instrument to assess organizational readiness for knowledge translation in healthcare organizations: the OR4KT. Int J Health Policy Manag.

[12] Gagnon MP, Attieh R, Ghandour el K (2014). A systematic review of instruments to assess organizational readiness for knowledge translation in health care. PLoS One.

[13] Hamilton S, McLaren S, Mulhall A (2007). Assessing organisational readiness for change: use of diagnostic analysis prior to the implementation of a multidisciplinary assessment for acute stroke care. Implement Sci.

[14] Pyone T, Smith H, van den Broek N (2017). Implementation of the free maternity services policy and its implications for health system governance in Kenya. BMJ Glob Health.

[15] Ritchie J, Lewis J, Nicholls CM, Ormston R. Qualitative Research Practice: A Guide for Social Science Students and Researchers. London: SAGE Publications; 2013.

[16] Baez-Carmago C, Jacobs E. A Framework to Assess Governance of Health Systems in Low Income Countries. Basel, Switzerland: Basil Institute on Governance; 2011.

[17] Siddiqi S, Masud TI, Nishtar S (2009). Framework for assessing governance of the health system in developing countries: gateway to good governance. Health Policy.

[18] Meessen B. An Institutional Economic Analysis of Public Health Care Organisations in Low-Income Countries. Louvain-la-Neuve: Université Catholique de Louvain; 2009.

[19] North DC. Institutions, Institutional Change and Economic Performance. Cambridge: Cambridge University Press; 1990.

[20] Marais DL, Petersen I (2015). Health system governance to support integrated mental health care in South Africa: challenges and opportunities. Int J Ment Health Syst.

[21] Petersen I, Marais D, Abdulmalik J (2017). Strengthening mental health system governance in six low- and middle-income countries in Africa and South Asia: challenges, needs and potential strategies. Health Policy Plan.

[22] Holt DT, Armenakis AA, Harris SG, Feild HS (2007). Toward a comprehensive definition of readiness for change: a review of research and instrumentation.

[23] Weiner BJ (2009). A theory of organizational readiness for change. Implement Sci.

[24] Gagnon MP, Labarthe J, Légaré F (2011). Measuring organizational readiness for knowledge translation in chronic care. Implement Sci.

[25] Saldana L, Chapman JE, Henggeler SW, Rowland MD (2007). The organizational readiness for change scale in adolescent programs: criterion validity. J Subst Abuse Treat.

[26] Lehman WE, Greener JM, Simpson DD (2002). Assessing organizational readiness for change. J Subst Abuse Treat.

[27] Bertone MP, Meessen B (2013). Studying the link between institutions and health system performance: a framework and an illustration with the analysis of two performance-based financing schemes in Burundi. Health Policy Plan.

[28] Mitchell A, Bossert TJ (2010). Decentralisation, governance and health-system performance: ‘where you stand depends on where you sit’. Dev Policy Rev.

[29] Aoki M. Toward a Comparative Institutional Analysis. Cambridge, Massachusetts: MIT Press; 2001.

[30] Erchick DJ, George AS, Umeh C, Wonodi C (2017). Understanding internal accountability in Nigeria’s routine immunization system: perspectives from government officials at the national, state, and local levels. Int J Health Policy Manag.

[31] Cleary SM, Molyneux S, Gilson L (2013). Resources, attitudes and culture: an understanding of the factors that influence the functioning of accountability mechanisms in primary health care settings. BMC Health Serv Res.

[32] Mirzoev T, Das M, Ebenso B (2017). Contextual influences on the role of evidence in health policy development: what can we learn from six policies in India and Nigeria?. Evid Policy.

[33] Pawson R, Tilley N (1997). Realistic Evaluation.

[34] World Bank. World Development Report 2004: Making Services Work for Poor People. World Bank; 2003.

[35] Alande JO. Role of Human Resource Management in Devolution of Counties in Kenya. Scientific Conference Proceedings; 2014.

[36] Booth D, Cammack DR. Governance for Development in Africa: Solving Collective Action Problems. Zed Books; 2013.

[37] Lipsky A, Mulaki A, Williamson T, Sullivan Z. Political Will for Health System Devolution in Kenya: Insights from Three Counties. Washington, DC: Health Policy Project; 2015.

[38] Barasa EW, Cleary S, Molyneux S, English M (2017). Setting healthcare priorities: a description and evaluation of the budgeting and planning process in county hospitals in Kenya. Health Policy Plan.

[39] Mikkelsen-Lopez I, Wyss K, de Savigny D (2011). An approach to addressing governance from a health system framework perspective. BMC Int Health Hum Rights.

[40] Greer SL, Vasev N, Jarman H, Wismar M, Figueras J. It’s the Governance, Stupid! TAPIC: A Governance Framework to Strengthen Decision Making and Implementation. Copenhagen, Denmark: European Observatory on Health Systems and Policies; 2019. 32045179

[41] Bandura A. Self-Efficacy: The Exercise of Control. New York, NY: W.H. Freeman/Times Books/ Henry Holt & Co; 1997.

[42] Bandura A (2000). Exercise of human agency through collective efficacy. Curr Dir Psychol Sci.

